# A model of self-directed learning in internal medicine residency: a qualitative study using grounded theory

**DOI:** 10.1186/s12909-017-0869-4

**Published:** 2017-02-02

**Authors:** Adam P. Sawatsky, John T. Ratelle, Sara L. Bonnes, Jason S. Egginton, Thomas J. Beckman

**Affiliations:** 1 Division of General Internal Medicine, Mayo Clinic, 200 First St SW, Rochester, MN 55905 USA; 2 Division of Hospital Internal Medicine, Mayo Clinic, Rochester, MN USA; 3 Robert D. and Patricia E. Kern Center for Science of Health Care Delivery, Mayo Clinic, Rochester, MN USA

**Keywords:** Adult learning theory, Graduate medical education, Self-directed learning

## Abstract

**Background:**

Existing theories of self-directed learning (SDL) have emphasized the importance of process, personal, and contextual factors. Previous medical education research has largely focused on the process of SDL. We explored the experience with and perception of SDL among internal medicine residents to gain understanding of the personal and contextual factors of SDL in graduate medical education.

**Methods:**

Using a constructivist grounded theory approach, we conducted 7 focus group interviews with 46 internal medicine residents at an academic medical center. We processed the data by using open coding and writing analytic memos. Team members organized open codes to create axial codes, which were applied to all transcripts. Guided by a previous model of SDL, we developed a theoretical model that was revised through constant comparison with new data as they were collected, and we refined the theory until it had adequate explanatory power and was appropriately grounded in the experiences of residents.

**Results:**

We developed a theoretical model of SDL to explain the process, personal, and contextual factors affecting SDL during residency training. The process of SDL began with a trigger that uncovered a knowledge gap. Residents progressed to formulating learning objectives, using resources, applying knowledge, and evaluating learning. Personal factors included motivations, individual characteristics, and the change in approach to SDL over time. Contextual factors included the need for external guidance, the influence of residency program structure and culture, and the presence of contextual barriers.

**Conclusions:**

We developed a theoretical model of SDL in medical education that can be used to promote and assess resident SDL through understanding the process, person, and context of SDL.

**Electronic supplementary material:**

The online version of this article (doi:10.1186/s12909-017-0869-4) contains supplementary material, which is available to authorized users.

## Background

Self-directed learning (SDL) is considered a component of physicians’ professional identities [1]. The Accreditation Council for Graduate Medical Education [2] requires that “residents and faculty members must demonstrate an understanding of their personal role in…attention to lifelong learning,” by developing skills and habits “to continuously improve patient care based on constant self-evaluation.” This “personal role” suggests that SDL is part of lifelong learning, and is an important competency for physicians to develop and maintain [3].

SDL originates from the adult education literature with Houle, Tough, and Knowles [4]. Knowles incorporated SDL into his adult learning theory by emphasizing “the learners’ self-concept of being responsible for their own decisions” and stating that “the most potent motivations [for learning] are internal pressures,” which contribute to “the transition from dependent to self-directing learners” [5]. Knowles [6] defined SDL as “a process in which individuals take the initiative, with or without the help of others, in diagnosing their learning needs, formulating goals, identifying human and material resources for learning, choosing and implementing appropriate learning strategies, and evaluating learning outcomes.” One review of SDL in medical education scholarship identified that many studies lacked a definition for SDL, highlighting that there is limited understanding of SDL and that clearer definitions and theories of SDL are needed to advance SDL research in medical education [7].

Starting with Knowles’ definition, theories of SDL have been developed to encompass three key components: process, personal attributes, and context. Brockett and Hiemstra [8] developed a Personal Responsibility Orientation model of SDL with two dimensions: SDL (process) and learner self-direction (motivation). Candy [9] subdivided these dimensions into four phenomena: personal autonomy, self-management, learner control in academic settings, and the individual, noninstructional pursuit of learning opportunities in the “natural societal setting.” Garrison [10] outlined three similar dimensions: self-management (task control), self-monitoring (cognitive responsibility), and motivation (entering and task). More recently, Hiemstra and Brockett [11] proposed that previous models underemphasized the effect of context on SDL and proposed a “Person, Process, Context” model, highlighting the equal importance of each of these three dimensions. They define *person* as the “characteristics of the individual,” such as “critical reflection, enthusiasm, life experience, motivation, and self-concept,” whereas *process* includes skills and abilities to carry out SDL [11]. This model added to their previous model the importance of context, which they defined as encompassing the “environmental and sociopolitical climate, such as culture, power, learning environment, political milieu…” [11]. This theoretical model highlights the complexity of SDL, incorporating the personal and contextual factors that affect the process of SDL.

Given the relevance of SDL to adult learning, understanding the application to medical education is critically important. Murad et al. [12] demonstrated that SDL was effective for knowledge acquisition in health professions education but identified that few studies reported SDL components consistent with Knowles’ definition. This suggests a misunderstanding of SDL in medical education and implies that clear definitions and the application of SDL theory can focus and clarify ongoing medical education scholarship in this area [7, 13].

Slotnick [14] studied SDL among physicians, which resulted in a 4-stage model of the process of SDL: scanning, deciding, learning, and gaining experience. Similarly, Li et al. [15] developed a model for the process of SDL in residency. Although these models outlined the process of SDL in medical education, they did not explore the components of people or context of SDL. To our knowledge, a comprehensive model of SDL in medical education—which incorporates process, person, and context—does not currently exist. Therefore, we sought to explore the person, process, and context of SDL during residency training.

## Methods

To build on existing theory and develop a framework of SDL in medical education, we used a constructivist grounded theory approach to explore the experience of SDL during internal medicine residency training at Mayo Clinic, Rochester, Minnesota, USA, from October 2014 to January 2015. Study investigators had experience in qualitative medical education research and residency education. To explore various experiences and to learn from the social interaction of participants, we collected data using focus groups. This study was approved by the Mayo Clinic Institutional Review Board. All participants provided informed consent.

The Internal Medicine Residency Program at Mayo Clinic includes 144 categorical residents and 24 preliminary residents. Sixty percent of the residents for this academic year were men. We invited all residents to participate in 1-h focus groups, which were moderated by an experienced facilitator (J.S.E.) who had no connection to the residency program. The primary investigator (A.P.S.) observed each session to provide initial data summaries. All residents who volunteered to participate were included in the study. We conducted 7 focus groups with 5–9 participants per group; each group discussion lasted 60 min. The focus group discussions were audio recorded and transcribed verbatim. Transcripts were de-identified before data analysis.

We developed the focus group guide through established methods, including a comprehensive review of the literature and review with a panel of residency faculty members [16]. Throughout data collection and analysis, we revised the interview guide to optimize saturation of themes within our theoretical model. We have included the focus group guide as a representation of possible questions, but emphasis may have been placed on different questions to ensure rich discussion and theory development (see Additional file 1, Box).

We used a constructivist grounded theory approach to develop a theoretical model for how residents engage in SDL [17]. We chose this approach because we wanted to develop a theoretical model of SDL that was unique to the residency learning environment, but was informed by previous SDL theory. We therefore used Hiemstra and Brockett's “Person, Process, Context” model as our theoretical lens to guide analysis and frame our research findings [11]. We analyzed data after each focus group discussion was transcribed. Using open-coding and writing analytic memos, we identified major themes. After the first two focus groups, team members categorized dominant themes to create axial codes, which were applied to all transcripts using NVivo (QSR International) [18].

We developed a theoretical model that was revised as new data were collected. Through constant comparison, we refined the theory until it had adequate explanatory power and was appropriately grounded in the experiences of residents. This process also allowed the study team to assess theoretical saturation, which was achieved after seven focus groups. To test the trustworthiness of our theory, we invited all 46 study participants to partake in one of two member check sessions, and 18 residents (39%) participated. In these sessions, we presented the theoretical model and discussed the process, personal aspects and contextual factors of SDL. Participants were given the opportunity to make comments and discuss the model. During these sessions, the study participants endorsed the nature of our findings and suggested minor changes to the model.

## Results

We conducted seven focus groups of 46 residents total: 20 postgraduate year-1 residents, 10 year-2 residents, and 16 year-3 residents. Thirty-one residents (67%) were men.

**Fig. 1:**
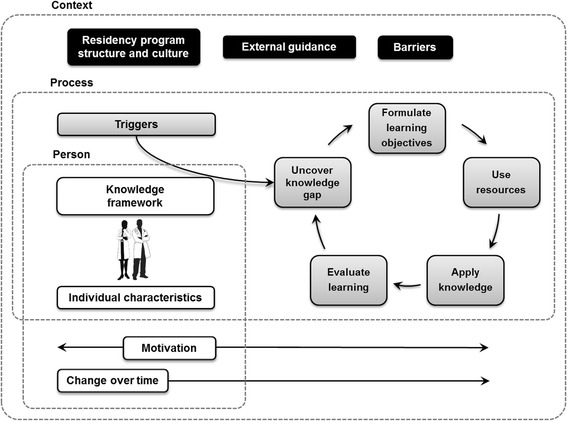
Theoretical Model of Resident Self-Directed Learning (SDL). This model highlights the person, process, and context of SDL in medical education, captured by the *dotted lines*. The *gray boxes* at the center represent the process of resident SDL. The *white boxes* represent personal factors that affect the process of SDL. The *black boxes* represent contextual factors that affect the process of SDL

We developed a broad theoretical model of resident SDL that encompassed the major themes within the categories of person, process, and context of SDL (see Fig. 1). The process of resident SDL is at the center of the model, beginning with a trigger for learning that acts on the resident’s knowledge framework to uncover a knowledge gap and stimulates the resident to formulate learning objectives, use resources, apply knowledge, and evaluate learning. This serves to build the resident’s knowledge framework and triggers additional learning, which makes SDL cyclical. The person of SDL includes motivations, individual characteristics, and change over time. The context of SDL includes external guidance, residency program structure and culture, and barriers. We will discuss each element below. (Quotations given are followed by the group number of the participant.)

### The Process of SDL

The center of the theoretical model contains the process of SDL practiced by residents (Figure, gray boxes); Table 1 contains additional supporting quotations. The starting point and main goal of SDL was building a knowledge framework required to be a physician. Residents described the requisite knowledge gained through training as “what I need to know to come out of residency [having] a broad and deep knowledge base” (group 2). On this knowledge base, residents developed a framework that supported comprehension of medical knowledge and application to patient care, until residents understand concepts “in depth” (group 3).

**Table 1:** Supporting quotations for themes in the process of resident SDL

Theme	Explanation	Participant quotations^a^
Knowledge framework	The main goal was building a knowledge *framework* required to be a physician	“In a perfect world I’d spend 2 h every day going through topics categorically and have this nice wide knowledge base and really have a good comprehensive understanding” (group 5).
		“To formulate a framework on my own that works for me … I was able to synthesize my own kind of format” (group 4).
Triggers	External events started the process of SDL	“Every time I have a patient that comes in with a problem that I don’t necessarily grasp, and I have to pull up whatever resource, that’s SDL, …I’m going to remember that framework that I’m starting to develop” (group 4).
		Faculty and senior residents who “ask the right questions” (group 2) can trigger SDL by making it “clear an area I’m weak in, and that’s the area I go try and fill the void. …So I like people asking me questions because that tells me where I’m weak and helps me get stronger in those areas” (group 4).
Uncover knowledge gap	The trigger uncovered a gap in the resident’s current framework	“SDL is the process of identifying your weaknesses and your goals for learning” (group 5).
		“It’s about filling in your own gaps of knowledge. …I’m taking care of a patient and they have [a problem] so you go read about it. … you’re filling in your own gaps of knowledge” (group 4).
Formulate learning objectives	The gap in knowledge led residents to identify objectives to fill the gap	“You get a concise and a clear question and say, ‘We’re trying to decide between these two drugs, which one is better?’ That’s a clear and concise question that’s directly [clinically] relevant and easy to answer” (group 4).
		“It’s such an open, broad, vast sea of stuff that I could be studying. Triaging what I should study, what order I should study it, how much time I should dedicate to it. The system, …I consider it to be SDL” (group 6).
Use resources	Based on their specific learning objective, residents chose appropriate resources	“Knowing what resources give you what information and what amount of time you’ll take to find it” (group 1).
		“So for example, when you’re trying to figure out how to treat a specific condition, a well-written review article can be very high yield. …I’ve had to go through a lot of trial and error to find out what resources I like for what topics and in what situations and I’ve had some guidance” (group 3).
		“A lot of this learning is not so much learning the topic but learning where to find information, how to access the right information at the right time, and what resources are available to us. …Those things are much more important to learn” (group 2).
Apply knowledge	Residents applied the knowledge gained through SDL	“If I read something and I don’t apply it anywhere for a few months, then it won’t stay with me, but applying it clinically and seeing it in a patient, making some difference with what you learned, is a very important factor in making it stay with you” (group 7).
		“I’ve found that I learn the best when I have to teach someone about something…when I have to actually read and understand everything fully so I can teach it to others” (group 7).
Evaluate learning	Residents used self-reflection and external feedback to evaluate their learning through SDL	“You need some external assessment; it’s really hard to assess yourself. You definitely need some external evaluation of your performance because you’re not objective about yourself” (group 4).
		“I don’t really feel convinced that I’ve learned anything until I encounter the same scenario again and feel more comfortable with it…or if you feel like you’re thinking about other things than you would have the first time around, those are some of the clues that make me feel like I’ve learned something” (group 3).
		“I didn’t realize I learned everything first year until I got an intern second year. … You always just feel like you are struggling to stay afloat. But when you get someone below you, that’s when I actually found out that it was working” (group 3).

*Abbreviation*: *SDL* self-directed learning

^a^Quotations given are followed by the group number of the participant

Triggers for SDL were external events that exposed gaps in the resident’s current knowledge framework. Triggers arose when residents were “presented with a new unfamiliar scenario” (group 1), like “when a question comes up with the care of a patient” (group 6). Triggers included patient care, clinical teaching, peer interaction, media reports, email notifications, and preparation for examinations. Once the gap in knowledge was exposed, residents identified specific learning objectives. Objectives often took the form of a specific clinical question, and residents identified several objectives for any trigger. Residents triaged objectives by prioritizing objectives that pertained to “common conditions” and that will “change my practice” (group 1).

To accomplish their learning objectives, residents sought resources, including clinical summaries, journal articles, Internet searches, colleagues, and faculty. Resource selection was influenced by the objective, and residents learned which resources helped achieve different types of objectives, searching for the most high-yield resources. Once a learning objective was achieved, the knowledge or skill was applied to the SDL trigger, a critical step in solidifying knowledge and evaluating the learning process.

Residents used self-reflection and external assessment to evaluate their learning. Self-reflection was often aided by external cues or feedback. External cues included knowledge application, comfort with patient care, efficiency, performance on clinical questions, and gauging themselves against their peers. Feedback came through faculty evaluation and performance on examinations (eg, In-Training Examination). Although residents sought external feedback, sometimes self-evaluation was based on a feeling: “I don’t know, to me it’s just a gut feeling. I know I’ve read enough, and if I read more, it’s just going to be useless” (group 2). At the same time, there was another sentiment: “It’s part of our profession…I can’t imagine getting to a point where I would say I’m totally comfortable” (group 6). Self-evaluation drove future learning, thereby creating a continuous cycle of SDL.

### The Person of SDL

Residents described multiple personal aspects of SDL, including their motivations, individual characteristics, and their change in approach over time.

#### Motivations

Several types of motivation moved residents through the process of SDL. The foundational motivation for SDL was intrinsic: “I equate [SDL] to intrinsic learning; it’s your own intrinsic motivation to learn outside of a defined curriculum” (group 4). Extrinsic motivation was also important for SDL during residency training: “Extrinsic motivators are very good, because there are certain things that I’m not that interested in intrinsically” (group 2).

Intrinsic motivations included personal interest, curiosity, enjoyment of learning, competence, personal responsibility, improved patient care, and professional identity formation (see Table 2). Residents also described emotional motivations, including fear of “looking stupid,” personal connections to a topic area, personal mistakes, and the need for self-preservation. Previously successful SDL was a powerful motivator: “there are a few moments that I can pinpoint…a case where it was almost palpable, where you started to dig into the details, and you discovered a linchpin that made everything flow together, and you knew exactly what was going on…at a very deep level that’s what keeps me going” (group 7). These “aha moments” (group 1) made SDL enjoyable and drove future learning. Additionally, residents were extrinsically motivated by patients, peers, faculty members, and examinations.

**Table 2:** Motivations for resident SDL with supporting quotations

Motivation	Participant quotations^a^
Personal interest	“It’s unlikely to come up on the board exam, but I still think it’s interesting so I’ll read about it, but that [is one of the] main things I consider as SDL” (group 1).
Curiosity	“I find that a lot of my SDL is a result of curiosity. It’s usually triggered by a patient encounter that makes me raise a question, and I keep probing until there comes a point when it gets uninteresting and I don’t have questions anymore” (group 6).
Enjoyment of learning	“The true essence of SDL is enjoyment. If you’re learning something without knowing that you’re learning it, then it’s probably SDL because you’re doing it without even thinking about it” (group 7).
Patient care	“Am I here because I like to be called a doctor or am I here because I want to know how to take care of patients the best I possibly can? I think that makes the biggest difference between SDL and doing the bare minimum” (group 6).
Competence	“At the end of the day, when you sit in a room with a patient, how competent are you” (group 6)? “It’s important to demonstrate competence in areas that you may not be so interested in so that you can still provide excellent care” (group 4).
Personal responsibility	“Being in the position where I had no safety net, I realized that only I could help in the situation, and so immediately I became more resourceful than I typically would have been in a situation like that, in how I perused resources and created an initial therapy plan. That was very instructional because when you’re put in that position you become more resourceful than you think you’re capable of, and to me that was like the crux of SDL” (group 6).
Identity formation	“Once you start figuring out your specific niche, you become more interested in that pathology and literature, and it’s interesting because your peers will come to you and ask about specific cases. It motivates you to really be on top of the area in which you’re going” (group 3).
Fear of looking stupid	“I’m afraid of looking dumb in front of the med students…patients…yeah, including yourself…there’s a constant fear of looking dumb” (group 5).
Emotional connection	“SDL is important when we’re emotionally tied to a specific topic. If we have a family member who is struggling from a specific illness, we might have a self-directed drive to learn more about that, or if we have an emotional connection to a patient we might go deeper just because there’s an emotional connection” (group 2).
Self-preservation	“When I have a rough day, I go back at the end of the day and I’m like, ‘Man why was I slogging through everything, why was it such a pain?’ Then I go, ‘Well, I didn’t know this.’ I should look that up so that next time I can have that discussion much more easily, and that’s one of the ways in which I drive myself to do SDL” (group 1).
Faculty inspiration	“In terms of motivation from consultants, you meet so many world-famous leaders in fields on a daily basis, and that’s really inspirational for me. Like the people around me really motivate me, and [faculty members] are a big part of that” (group 3).
Social pressure	“To be honest with you, for me it’s a lot of [my peers]. I mean, these guys are always learning, and I feel like if I don’t, I’ll be left behind” (group 3).
Examinations	“All the residents care about is, ‘Is this coming up on my boards, is this coming up on Step 3?’ I feel that is really big” (group 4).
Mistakes	“I find that I learn the best from my own mistakes. If I did something and I was like, ‘Oh crap, I screwed up,’ that stays with me and I become the unofficial expert in that thing because I messed it up” (group 4).
Previous success	“The moments are fleeting, but when they do occur it’s fun, but when you see a patient and you think about it more and you’re like, ‘I’ve seen this before and I know this,’ and you figure it out. That’s what makes it enjoyable—the aha moments” (group 1).

*Abbreviation*: *SDL* self-directed learning

^a^Quotations given are followed by the group number of the participant

Residents also discussed factors that reduced motivation to pursue SDL. First, unrealistic expectations “can impede your desire to participate [in SDL] because you don’t feel you can accomplish that goal” (group 3). Second, when residents experienced little autonomy or responsibility for patient care, “that doesn’t help our SDL when the [faculty members] are not [involving] the residents [in patient care]” (group 7). These factors eroded motivation for SDL.

#### Individual characteristics

Residents discussed several individual characteristics that affected the process of SDL. First, residents have different levels of confidence with SDL, which affected how they approached SDL: “Everybody comes into residency with varying levels of confidence regarding SDL, and they should teach you how to do SDL” (group 2). Second, residents identified variations in preferences that could influence SDL: “learning styles are important, because to some people SDL is more important than to others” (group 1). Personal styles affected how residents structured SDL: “There may be an element of personality that carries over into how you learn. Do you need it to be more structured or more free-flowing?” (group 6). The approach to choosing and using resources can also differ based on “styles of learning…some people can picture things and other people learn in other ways” (group 7). Although individual characteristics affected how and when residents participated in SDL, it still followed the same basic process.

#### Change over time

Residents’ approach to SDL changed over the course of their training, as residents developed confidence in SDL and sophistication in their knowledge framework: “As you progress in medicine, you’re able to deal with the nuance better, and that’s where experience comes into play” (group 7). A more advanced framework had smaller gaps and led to more specific learning objectives. As learning objectives changed, residents used different resources, progressing from textbooks to clinical reviews to original research. Over time, residents become more confident in their ability to identify and use the appropriate resource for a given objective: “it gets more efficient because you find different resources for different situations” (group 7).

### The context of SDL

Residents discussed multiple contextual aspects affecting SDL, including the need for external guidance for SDL, the influence of residency program structure and culture, and contextual barriers.

#### External guidance

Although the process of SDL was characterized by internal motivation and choice about learning, residents also identified the need for external guidance (see Table 3). Residents described guidance for SDL as different from “other-directed learning, learning that is constructed by others…when we’re seeing our patients, at home thinking at night, investigating what we find interesting…that really is SDL” (group 1). They identified sources of guidance for SDL, including the residency curriculum, individual faculty members, peers, patients, and examinations. External guidance helped focus SDL and provided support for continued learning, and residents saw the benefit of being provided with a structure “to guide your learning, and then you can get feedback on how your search went and how you can do better next time” (group 4) and “maximize your self-directed gain” (group 5).

**Table 3:** External guidance for resident SDL with supporting quotations

Step in SDL process	Type of external guidance	Participant quotations^a^
Framework	External sources helped provide a framework for learning	“For everything that’s key, they need to provide a framework and the key things that you have to know. Then provide the resources for those that are interested in going deeper” (group 4).
Uncover knowledge gaps	External sources uncovered residents’ knowledge gaps	“I like people asking me questions, and I like people giving me a hard time because that tells me where I’m weak, and that helps me get stronger in those areas” (group 4).
Formulate learning objectives	External sources helped identify and focus learning objectives	“Sometimes they help us identify an objective, you know, something to learn” (group 1).
		“Sometimes when you’re doing SDL and you don’t have something to guide you, it’s very easy to miss out on what is really important. … If someone with clinical experience were teaching you, they could say the main things here are X, Y, and Z, but it’s easy to miss out on those things when you’re reading on your own” (group 4).
Use resources	Residents used people as a primary resource	“There are guidelines, but their 40 years of working has given them experience, and having that explanation is very helpful so we can understand from their experience what setting you would use this” (group 2).
		“Faculty can overrefer you to resources instead of just telling you theanswer” (group 3).
	External sources provided resources	“It can be really helpful; I’ve had consultants that say this review article is really good for this topic” (group 1).
	External sources taught how to use resources	“It’s good to know what’s available for resource and if someone tells you that ahead of time, then you can already sort that out without having to figure it out yourself. It’s nice to have the program say, ‘These are the resources’” (group 1).
Apply knowledge	Residents applied knowledge outside of themselves	“The main one for me by far is having had the chance to apply this in clinical practice” (group 7).
Evaluate learning	Residents sought feedback from multiple external sources	“We have the In-Training exams every year. They give you a percentage of how many questions you got right and wrong and you can compare yourself to your peers” (group 1).
		“The reason we are here is to get feedback to make ourselves better and I’m really appreciative of those people who take that step” (group 4).

*Abbreviation*: *SDL* self-directed learning

^a^Quotations given are followed by the group number of the participant

#### Residency program learning environment

Residency program learning environment, including the structure and culture, also influenced SDL. Residents recognized that the residency program needed to provide “time and resources to be able to pursue interests” (group 2) because “every rotation is a good time to be adding more nuggets of wisdom on SDL” (group 2). Time was necessary because “to really understand a subject, I have to go back when I’m not stressed out, where I have time to sit and actually think about what I’m reading” (group 6). Residency programs can also promote SDL by attempting to “cultivate a culture of learning among the residents …. SDL should be something that we start from day one of intern year, teaching you how to do it and making it an expectation” (group 3). Culture also related to social aspects of SDL, including motivation (“keeping up with the colleagues” [group 2]), evaluation (“a lot determined by what your peers know” [group 5]), and social identity (balance between trying to “make your own area and claim your stake” and trying to “fit in” [group 4]).

#### Barriers

Barriers to SDL were mostly contextual. A major barrier to SDL was having adequate time in the setting of competing demands: “The nature of being a resident is there are millions of things on every patient that you could look up, and we don’t have the time because we’re busy” (group 3). The main demand on time was the balance between patient care and SDL: “You can’t choose when the patient comes in; you try to fit in learning, and the fact that your schedule is crammed impedes how much learning you can do” (group 1). Obligations, including research and other learning opportunities, were barriers to SDL. Maintaining personal well-being could detract from SDL: “if we have some time, that’s not what we want to spend our time doing, in order to have a balance and sanity” (group 5). Striking this balance was discussed as a barrier to SDL but was seen as necessary “to not get burned out, because if you get burned out then there’s nothing much you can do …. That ties into how you manage your time and energy level” (group 1).

Difficulty with any step of the process also served as a barrier to SDL overall. For example, residents who have difficulty identifying knowledge gaps, translating them into learning objectives, and quickly identifying appropriate resources may struggle with SDL: “I don’t know what my weaknesses are to say I have these goals for learning” (group 4) or “there’s so much I don’t know that my list of things to read is so ridiculously long that I have no idea where to start” (group 2).

## Discussion

We present a broad theoretical framework of SDL in medical education that includes aspects of process, person, and context. The process begins when external triggers expose gaps in residents’ knowledge framework, which stimulates them to engage in a cycle of formulating learning objectives, identifying learning resources, applying knowledge to clinical problems, evaluating their learning, reinforcing their growing framework, and possibly triggering further SDL. The person aspect, which includes a complex interplay of individual comfort with and motivation for SDL, develops over the course of residency training. Finally, context, encompassing individual faculty members and residency structure and culture, affects the process of SDL.

This study builds on previous theoretical models for SDL in medical education, contextualizing broader theory of SDL [11] to the residency training environment. In contrast to practicing physicians [14], residents spend less time planning and react to clinical problems in real time. This may reflect the evolution of learning in the online environment, in which information is immediately available. We also identified similar personal and contextual barriers to SDL as those identified by Li et al. [15, 19] and have integrated them into our conceptual model. Additionally, our findings are congruent with previous work suggesting the need for external guidance for SDL; we have also included these contextual factors in our model [20].

We utilized previous theoretical models from general education research to enhance our understanding of SDL during residency [8–11]. There is a growing understanding that SDL is influenced by context, and there has been a call for more qualitative inquiry into the role of context and SDL in medical education [21]. This study underscores distinct triggers for learning and the value of applying knowledge in medicine. We elucidate how personal and contextual factors affect SDL among residents and demonstrate how these factors influence learning. For example, contextual factors, like the residency learning environment, including culture and program expectations, can affect both the process of and personal motivation for SDL. Personal growth can influence the process of SDL as residents progress through their training. Context can greatly affect the process and person aspects of SDL.

SDL is felt to be integral to resident learning and identity formation [1]. Residency programs have implemented individualized learning plans to augment SDL [22–24]. Despite these efforts, residents have identified difficulty with SDL and desired guidance from faculty [19, 20, 25]. We are hopeful that our model—which exposes important contextual factors and provides an overarching definition of SDL within graduate medical education—will address existing limitations by providing residents with an improved understanding of SDL and providing faculty with better insights regarding their roles in helping residents learn.

This study has some limitations. It was conducted at a single institution, which may limit transferability to other settings. Additionally, these findings may not describe SDL in different environments, such as surgical subspecialties or undergraduate medical education. Future research should focus on how SDL differs across the spectrum from undergraduate to continuing medical education. Finally, these data represent residents’ perceptions of SDL, which should prompt future research regarding observed practices.

## Conclusions

Guided by existing SDL theory, we developed a theoretical model of resident SDL that highlights the personal (eg, motivations, individual characteristics) and contextual (eg, residency program structure, external guidance, and barriers) factors that affect the process of SDL. This model may improve understanding of SDL in graduate medical education, allow residency programs to achieve an appropriate balance between SDL and other learning opportunities, and provide a framework for future research on developing instruments to assess SDL.

## Additional file

Additional file 1: Box. Interview guide for the study entitled: “Self-Directed Learning in Internal Medicine Residency: A Qualitative Study”. (DOC 45 kb)

## Abbreviation

SDL: Self-directed learning
