# Supplementary material for: A model of self-directed learning in internal medicine residency: a qualitative study using grounded theory
Source: BMC Med Educ. 2017 Feb 2;17:31. doi: 10.1186/s12909-017-0869-4 (PMC5288975; doi:10.1186/s12909-017-0869-4)
Supplement: Additional file 1: Box. — Interview guide for the study entitled: “Self-Directed Learning in Internal Medicine Residency: A Qualitative Study”. (DOC 45 kb) [file 12909_2017_869_MOESM1_ESM.doc]

**Additional Files**

**Box. Self-Directed Learning in Internal Medicine Residency: A Qualitative Study**

| **Focus Group Guide** |
| --- |
| 1. Opening Question: 2. Tell us your name, where you went to medical school, and what comes to mind when you think about **self-directed learning**? 3. Transition Questions: 4. How would you define self-directed learning?   [Prompt] What are the main components of self-directed learning?  [Prompt] What are the main skills needed for self-directed learning?   1. What are the main motivators for self-directed learning?   [Prompt] How do you assess your own learning goals?  [Prompt] How do you come up with a learning plan?   1. What are the main resources you use for self-directed learning?   [Prompt] How do you identify sources for self-directed learning?   1. How do you know that you learned something? How do you track your learning? 2. Key Questions: 3. What are some common missed opportunities for self-directed learning, where it should have occurred but didn’t? What was the cause for those missed opportunities?   [Prompt] What are the main barriers to self-directed learning?  [Prompt] How do faculty support or hinder your self-directed learning?  [Prompt] How have you attempted to overcome those barriers?  [Prompt] What could be done to help overcome those barriers?  [Prompt] If patient care is a barrier, probe about how they balance patient care as the trigger for SDL and a barrier for SDL.   1. We have been discussing the skills necessary to perform self-directed learning (identifying learning needs, formulating learning goals, identifying and evaluating resources for learning, selecting and implementing learning strategies, evaluating learning outcomes). How have you been trained in these skills?   [Prompt] How does the residency program train you in those skills?  [Prompt] How do you rate yourself with those skills?   1. What, if any, help would you like to have from the residency program with to help support self-directed learning?   [Prompt] How can your staff consultants support self-directed learning?  [Prompt] What kinds of topics would be best suited for self-directed learning?  [Prompt] When would be the best time to incorporate teaching on self-directed learning into the residency curriculum?   1. Ending Questions: 2. If you had a chance to give advice to the program director on this topic, what advice would you give? Of all the things we discussed, what is the most important to you? (Can ask this of all participants) 3. Anything else you would like to add? |
